# Preoperative consolidation‐to‐tumor ratio is effective in the prediction of lymph node metastasis in patients with pulmonary ground‐glass component nodules

**DOI:** 10.1111/1759-7714.13899

**Published:** 2021-02-25

**Authors:** Yi‐Chung Chen, Yi‐Han Lin, Hung‐Che Chien, Po‐Kuei Hsu, Jung‐Jyh Hung, Chien‐Sheng Huang, Chih‐Cheng Hsieh, Wen‐Hu Hsu, Han‐Shui Hsu

**Affiliations:** ^1^ Division of Thoracic Surgery, Department of Surgery Taipei Veterans General Hospital Taipei Taiwan; ^2^ Division of Thoracic Surgery, Department of Surgery Min‐Sheng General Hospital Taoyuan Taiwan; ^3^ Institute of Emergency and Critical Care Medicine National Yang‐Ming University School of Medicine Taipei Taiwan; ^4^ Department of Surgery New Taipei City Hospital, Sanchong New Taipei City Taiwan

**Keywords:** consolidation‐to‐tumor ratio, ground‐glass opacity, lung cancer, positron emission tomography/computed tomography

## Abstract

**Background:**

Preoperative positron emission tomography/computed tomography (PET/CT) is recommended as a guideline for staging of lung cancer. However, for patients with pulmonary ground‐glass opacity (GGO) nodules who are supposed to have a relatively low risk of incidence of lymphatic metastasis, it remains uncertain whether PET/CT is more effective than consolidation‐to‐tumor ratio (CTR) in the prediction of regional lymphatic metastasis.

**Methods:**

The data on patients who underwent surgery for lung cancer from 2011 to 2016 were collected retrospectively, which included CTR, results of PET/CT, and pathological characteristics. The patients who had undergone preoperative PET/CT were identified to find the risk factors for lymphatic metastasis. A receiver operating characteristic (ROC) curve and multiple logistic regression was utilized to clarify the predictive value of CTR and main tumor maximal standardized uptake value (SUVmax).

**Results:**

Among 217 patients who had PET/CT before lobectomy, chest computed tomography revealed that 75 patients had CTR greater than 62%. The patients with lymphatic metastasis were shown to have higher CTR and higher main tumor SUVmax. Multiple logistic regression showed that younger age (<60 years), higher main tumor SUVmax on PET/CT, and greater CTR were independent predictive factors for lymphatic metastasis. The area under the ROC curve was comparable, 0.817 for CTR, and 0.816 for main tumor SUVmax.

**Conclusions:**

The present study revealed that CTR was not inferior to main tumor SUVmax considering the predictive power for lymphatic metastasis preoperatively in lung cancer patients with a GGO component. PET/CT might not be necessary preoperatively in selected patients.

## Graphical abstract

For lung cancer patients with a ground‐glass opacity (GGO) component, CTR was not inferior to main tumor SUVmax for the prediction of lymph node metastasis in a preoperative setting. PET/CT might not be necessary preoperatively in selected patients at low risk of incidence of lymph node metastasis.

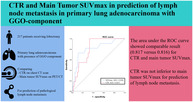

## INTRODUCTION

Lung cancer is one of the most prevalent cancers in the world. It initially has no obvious symptoms but may develop into distant metastases with an extremely high mortality rate. Air pollution, smoking and family history are possible causes of lung cancer.^1^ With the development of low‐dose chest computed tomography (LDCCT) screening in recent years, people exposed to risk factors are encouraged to undergo lung cancer screening.^2^, ^3^ According to the National Comprehensive Cancer Network (NCCN) guidelines, surgical resection is the standard treatment for early lung cancer, in view of its relatively low recurrence rate after surgical resection and expectable long‐term survival.^2^, ^4^

For patients with lung cancer, positron emission tomography/computed tomography (PET/CT) and endobronchial ultrasound (EBUS) are widely used to detect the possibility of mediastinal lymphatic metastasis or distant metastasis.^5^ These examinations can effectively help clinicians distinguish whether lymph node metastasis is present or not. According to a previous study, the mediastinal lymphatic metastasis rate of lung cancer can reach as high as 25.8%.^6^ While the presence of mediastinal lymphatic metastasis is the watershed of surgery or not for patients with lung cancer,^2^ radical lymph node dissection during surgery is recommended in order to achieve more accurate staging.^7^, ^8^ For patients with an established diagnosis of mediastinal lymphatic metastasis, induction therapy should be considered prior to surgery.^2^

Recent reports have demonstrated some cases of early lung cancer presenting as ground‐glass opacities (GGOs) on computed tomography which were less invasive.^9^ Other studies have confirmed a relatively low lymph node metastasis rate in these GGO‐type lung cancers.^10^, ^11^ For these patients, preoperative PET/CT might not be necessary. In this study, we aimed to investigate whether PET/CT was essential for patients with GGO‐type lung adenocarcinoma, and whether the consolidation‐to‐tumor ratio (CTR) on chest CT was effective in the prediction of lymph node metastasis in lung cancer.

## METHODS

The clinical data of patients who had undergone surgery for primary lung cancer from 2011 to 2016 were collected retrospectively. This data included patient age, gender, tumor size, histology, staging, lymph node status, harvested lymph node stations/numbers, CTR, and the results of PET/CT scan. Tumor staging was determined according to the International Association for the Study of Lung Cancer, eighth edition of the TNM classification for non‐small cell lung cancer (NSCLC).^12^ Exclusion criteria were patients with pure solid nodules, nonadenocarcinoma lung cancer, and patients who had undergone sublobar resection. The study was approved by the Institutional Review Board of Taipei Veterans General Hospital (No: 2020‐03‐008AC).

Chest CT images were analyzed with a high frequency algorithm and examined with a window level of −550 Hounsfield unit (HU) and a window width of 1600 HU, as the lung window. The mediastinal window was defined as window level of 40 HU and window width of 400 HU. The size of GGO was defined as the maximal diameter of the GGO part in the lung window, and the size of solid part was defined as the maximal diameter of the solid part in the mediastinal window. CTR was defined as the maximal solid part diameter divided by the GGO part diameter.

PET/CT examination was performed by intravenous injection of 2‐deoxy‐2‐(F‐18) fluoro‐D‐glucose (^18^F‐FDG) to obtain CT images, followed by PET scans an hour later. A maximal standardized uptake value (SUVmax) of the lymph nodes exceeding 2.5 was judged by the clinician as lymph node metastasis or reactive lymphadenopathy.

The surgical criteria were as follows: newly discovered GGO nodules were followed with a chest CT scan after three to six months, and then every 6–12 months afterwards. If the tumor size had increased by more than 20%, or the density of the solid part of the tumor had increased, surgery would be considered. Patients fulfilling the surgical criteria received PET/CT based on the clinical judgment of the surgeon. Patients with risk factors such as smoking history, family history of lung cancer, lymph node enlargement, or tumor with high CTR on chest CT, were more likely to receive PET/CT examination. Those without these risk factors tended to receive direct surgery.

Patients who underwent preoperative PET/CT were first analyzed to understand the risk factors for lymph node metastasis. Categorical data were reported as counts with percentages and compared with Pearson's chi‐squared test or Fisher's exact test. Continuous variables were given as mean ± SD and compared using two sample *t*‐test or Mann–Whitney U test. A *p‐*value <0.05 was considered statistically significant. Receiver operating characteristic (ROC) curves and sensitivity tests were used to find the optimal cutoff values for CTR and main tumor SUVmax. The degree of agreement of the two parameters was compared with Kappa coefficients. A multiple logistic regression model was utilized, adjusted for age, gender, and variables with *p*‐value <0.2 to further examine the significant factors identified. After preliminary analysis, this result was then applied to all patients undergoing lobectomy. In the two‐stage analysis, we hoped to determine the risk factors of lymph node metastasis for pulmonary adenocarcinoma with a GGO component, and evaluate the predictive power of preoperative PET/CT and CTR on chest CT. Data analysis was performed using IBM SPSS software (IBM Corp. Released 2017. IBM SPSS Statistics for Windows, Version 25.0. IBM Corp).

## RESULTS

**FIGURE 1:**
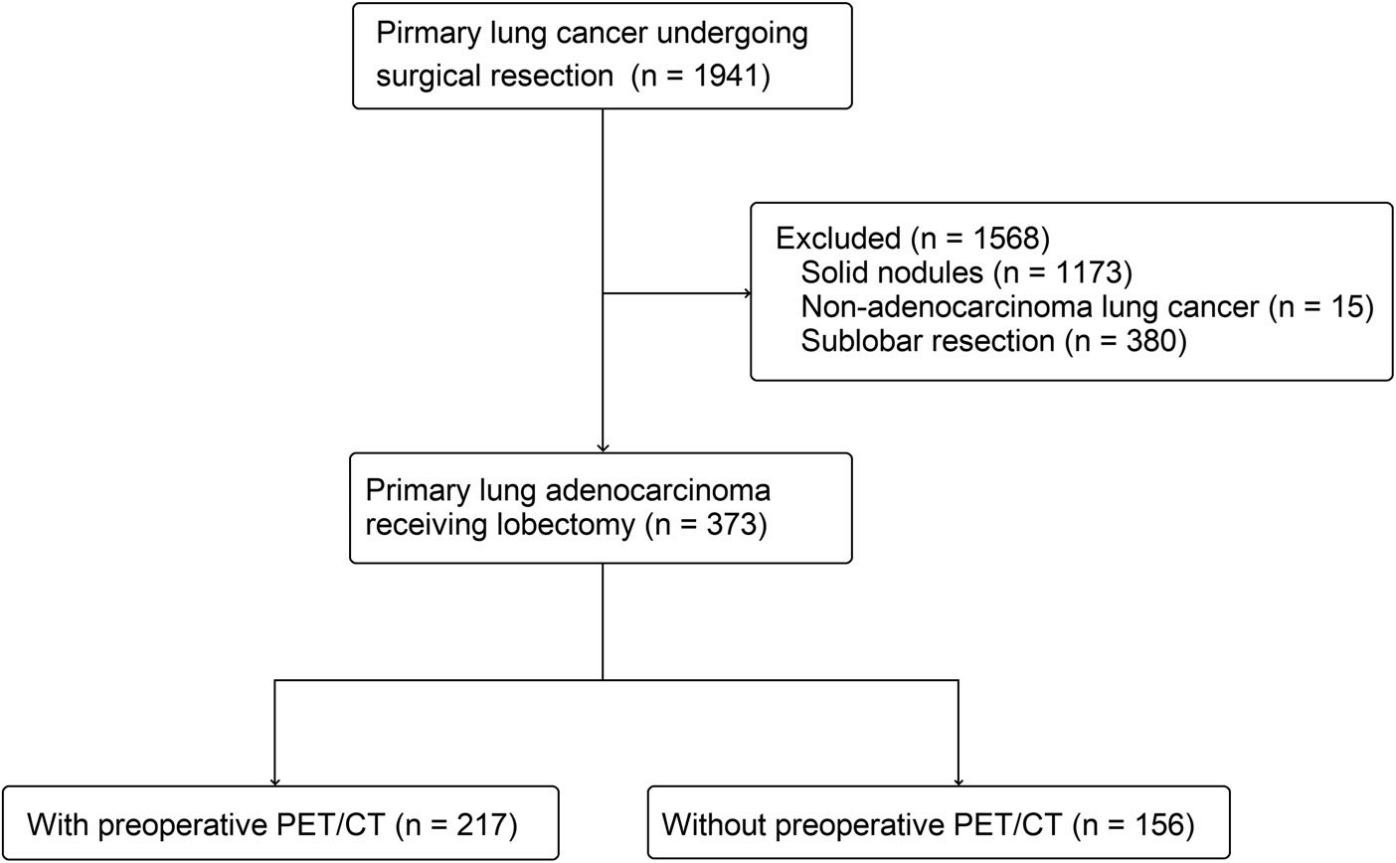
Flow diagram for patient selection (positron emission tomography/computed tomography [PET/CT])

A total of 1941 patients underwent lung resection surgery during the period from 2011 to 2016. After exclusion of pure solid nodules, nonadenocarcinoma lung cancer, and patients who had undergone sublobar resection, 373 patients were enrolled in the analysis (Figure 1). The demographic data are shown in Table 1. Among these patients, 217 patients had preoperative PET/CT and 156 patients had no preoperative PET/CT. The mean follow‐up period was 43.3 months. In the patients who underwent preoperative PET/CT, 10 were diagnosed as having clinical mediastinal lymph node metastasis: two had a mediastinoscopic lymph node biopsy with a negative result, while the other eight patients underwent surgical resection directly after panel discussion.

**TABLE 1:** Demographic data for patients receiving lobectomy for lung adenocarcinoma

Variables	Data
Age (years, mean ± SD)	60.9 ± 9.8
Gender (%)	
Male	144 (38.6)
Female	229 (61.4)
Smoking status (%)	68 (18.2)
Family history (%)	43 (11.5)
Tumor size (cm, mean ± SD)	
GGO part	2.09 ± 0.93
Solid part	0.97 ± 0.88
CTR (%, mean ± SD)	40.7 ± 30.7
PET/CT (%)	
N0	198 (53.1)
N1	9 (2.4)
N2	10 (2.7)
Not performed	156 (41.8)
Harvested lymph node (mean ± SD)	
Stations	4.4 ± 1.5
Number	14.8 ± 8.2
Pathological subtype (%)	
Lepidic‐predominant	156 (41.8)
Acinar‐predominant	171 (45.8)
Papillary‐predominant	32 (8.6)
Micropapillary‐predominant	8 (2.2)
Solid‐predominant	6 (1.6)
Pathological T stage (%)	
Tis	24 (6.4)
T1mi	39 (10.5)
T1a	29 (7.8)
T1b	61 (16.4)
T1c	39 (10.5)
T2a	169 (45.3)
T2b	10 (2.7)
T3	2 (0.5)
Pathological N stage (%)	
N0	356 (95.4)
N1	9 (2.7)
N2	7 (1.9)

Abbreviations: CTR, consolidation‐to‐tumor ratio; GGO, ground‐glass opacity; PET/CT, positron emission tomography/computed tomography.

Based on pathological lymph node status, the patients were categorized into nodal negative (pN0, *n* = 206) and nodal positive (pN+, *n* = 11) groups for analysis (Table 2). More solid part of the tumor was observed in the pN+ group (1.86 ± 0.51 vs. 1.11 ± 0.88 cm, *p* = 0.005). A higher CTR ratio (75.4 ± 12.4% vs. 44.7 ± 29.3%, *p* = 0.001) and main tumor SUVmax (5.2 ± 3.3 vs. 2.2 ± 2.2, *p* < 0.0001) were also observed in patients with lymph node metastasis.

**TABLE 2:** Comparison based on pathological lymph node status in patients with PET/CT

Variables	pN0 (*N* = 206)	pN+ (*N* = 11)	*p*‐value
Age (years, mean ± SD)	62.3 ± 10.3	57.4 ± 5.4	0.120
Gender (%)			0.031
Male	82 (39.8)	8 (72.7)	
Female	124 (60.2)	3 (27.3)	
Smoking status (%)	43 (20.9)	2 (18.2)	0.831
Family history (%)	23 (11.2)	3 (27.3)	0.110
Tumor size (cm, mean ± SD)			
GGO part	2.26 ± 0.94	2.49 ± 0.71	0.427
Solid part	1.11 ± 0.88	1.86 ± 0.51	0.005
CTR (%, mean ± SD)	44.7 ± 29.3	75.4 ± 12.4	0.001
Main tumor SUVmax (mean ± SD)	2.2 ± 2.2	5.2 ± 3.3	<0.0001
PET/CT			0.056
N0	190 (92.2)	8 (72.7)	
N1	8 (3.9)	1 (9.1)	
N2	8 (3.9)	2 (18.2)	
Harvested lymph node (mean ± SD)			
Stations	4.5 ± 1.5	4.7 ± 1.7	0.679
Number	15.2 ± 8.2	17.0 ± 11.6	0.492
Pathological subtype (%)			0.003
Lepidic‐predominant	82 (39.8)	0	
Acinar‐predominant	100 (48.5)	8 (72.7)	
Papillary‐predominant	16 (7.8)	1 (9.1)	
Micropapillary‐predominant	4 (1.9)	0	
Solid‐predominant	4 (1.9)	2 (18.2)	
Pathological T stage (%)			0.094
Tis	6 (2.9)	0	
T1mi	18 (8.7)	0	
T1a	12 (5.8)	0	
T1b	31 (15.0)	1 (9.1)	
T1c	26 (12.6)	1 (9.1)	
T2a	106 (51.5)	7 (63.6)	
T2b	6 (2.9)	1 (9.1)	
T3	1 (0.5)	1 (9.1)	
Pathological N stage (%)			<0.0001
N0	206 (100)	0	
N1	0	7 (63.6)	
N2	0	4 (36.4)	

Abbreviations: CTR, consolidation‐to‐tumor ratio; GGO, ground‐glass opacity; PET/CT, positron emission tomography/computed tomography; SUVmax, maximal standardized uptake value.

**FIGURE 2:**
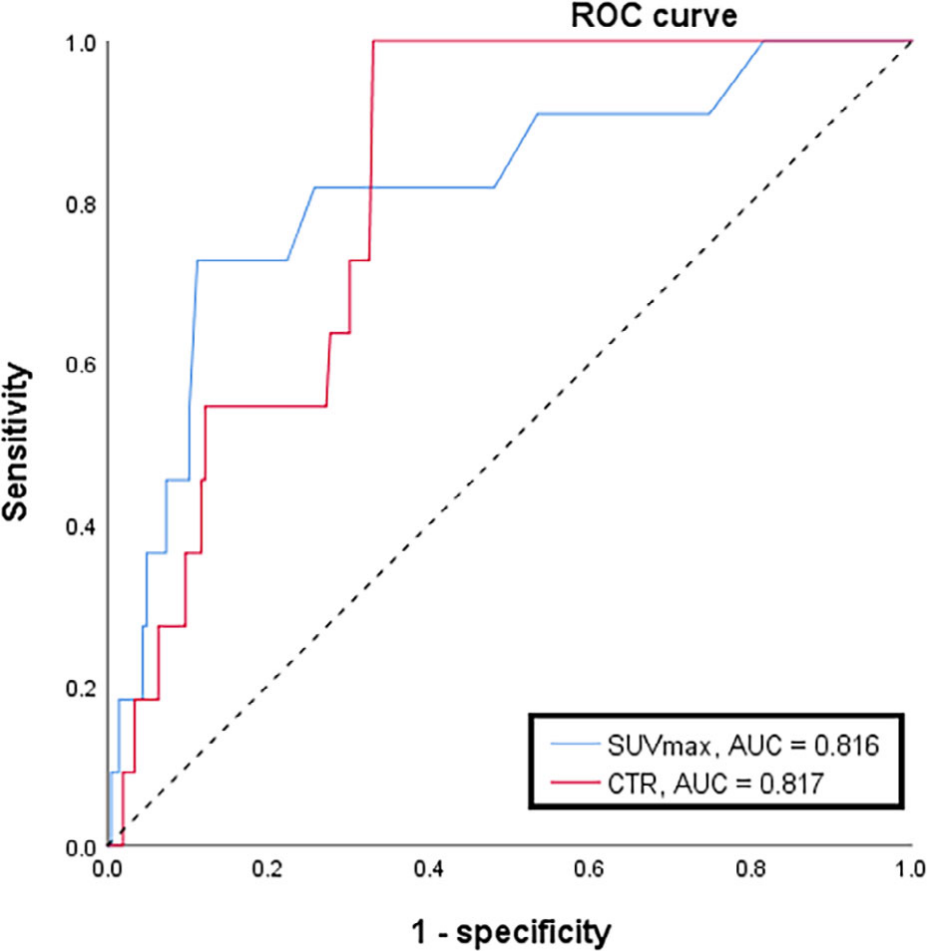
Receiver operating characteristic (ROC) curve for consolidation‐to‐tumor ratio (CTR) on chest computed tomography scan and main tumor maximal standardized uptake value (SUVmax) on PET/CT demonstrated a comparable area under the ROC curve, 0.816 for main tumor SUVmax and 0.817 for CTR

With the use of ROC curves and sensitivity tests, we determined a cutoff value of 62% for CTR, and 2.5 for SUVmax. For prediction of regional lymph node metastasis, the sensitivity was 72.7% for CTR on chest CT scan, and 27.3% for main tumor SUVmax on PET/CT. The area under the ROC curve was 0.817 for CTR, and 0.816 for main tumor SUVmax (Figure 2). The Kappa coefficient for CTR and main tumor SUVmax was 0.182 (*p* = 0.007). Two multiple logistic regression models were applied, both adjusting for age, gender, family history, and smoking history. CTR and main tumor SUVmax was analyzed separately in these two regression models due to collinearity. It showed that younger age (<60 years), higher main tumor SUVmax (≥2.5), and a greater CTR (≥62%) were independent predictive factors for lymph node metastasis (Table 3). These data indicated that, regarding the predictive power of regional lymph node metastasis, there was no significant difference between CTR and main tumor SUVmax.

**TABLE 3:** Multiple logistic regression for pathological lymph node metastasis in patients with preoperative PET/CT

Covariates	Adjusted OR	95% CI	*p*‐value	Adjusted OR	95% CI	*p*‐value
Age						
≥60 years old	1.0	‐	‐	1.0	‐	‐
<60 years old	8.614	1.649–45.006	0.011	13.793	2.390–79.611	0.003
Gender						
Female	1.0	‐	‐	1.0	‐	‐
Male	3.909	0.865–17.672	0.077	8.157	1.635–40.682	0.010
Family history						
Negative	1.0	‐	‐	1.0	‐	‐
Positive	3.312	0.598–18.337	0.170	4.299	0.739–25.017	0.105
Main tumor SUVmax						
<2.5	1.0	‐	‐			
≥2.5	14.832	2.837–77.548	0.001			
CTR						
<62%				1.0	‐	‐
≥62%				12.723	2.559–63.252	0.002

Note: Covariates including age, gender, and family history were controlled.

Abbreviations: CI, confidence interval; OR, odds ratio; PET/CT, positron emission tomography/computed tomography; SUVmax, maximal standardized uptake value.

Male gender was initially recognized as a significant factor for lymph node metastasis, but it showed an insignificant result in multiple logistic regression after controlling main tumor SUVmax. The characteristics of the patients with lymph node metastasis are described in Table 4. All these patients had CTR higher than 60%. Eight out of 11 patients were initially classified preoperatively as nodal negative.

**TABLE 4:** Tumor characteristics of patients with preoperative PET/CT examination and pathological lymph node metastasis

Gender	Tumor size (cm)	CTR (%)	SUVmax	Clinical N stage	Predominant subtype	Positive nodal stations
Male	1.3	62	11.8	2	Acinar	11 L
Male	2.6	62	1.6	0	Solid	12 R
Male	2.6	62	5.9	0	Acinar	10 L
Female	3.9	64	3.8	0	Acinar	10 R
Male	3	67	3.8	0	Solid	7 R
Male	1.6	81	2.5	0	Acinar	11 L
Male	3.1	81	5.2	1	Acinar	11 R
Female	2.3	83	4.2	0	Papillary	4, 10 R
Female	2.3	87	9.9	0	Acinar	10, 11 R
Male	2.2	91	6.4	2	Acinar	5, 11 L
Male	2.5	92	4.0	0	Acinar	2, 4, 7 R

Abbreviations: CTR, consolidation‐to‐tumor ratio; L, left; PET/CT, positron emission tomography/computed tomography; R, right; SUVmax, maximal standardized uptake value.

With a cutoff value of 62%, we divided the study cohort into two groups for comparison (Table 5). The patients with CTR ≧ 62% had both significantly larger GGO part (2.49 ± 0.86 vs. 1.91 ± 0.90 cm, *p* < 0.0001) and solid part size (1.90 ± 0.66 vs. 0.56 ± 0.60 cm, *p* < 0.0001). Significant differences in pathological subtype and stage were also noted. CTR < 62% was significantly associated with more patients at Tis, T1mi, and T1a stages (34.1% vs. 3.5%, *p* < 0.0001). In 115 patients with CTR ≧ 62%, 12 (10.4%) had lymph node metastasis, in contrast to five (1.9%) with lymph node metastasis in 258 patients with the CTR < 62% (*p* = 0.001). These findings indicated that patients with higher CTR ratio, especially when over 62%, were more likely to have lymph node metastasis.

**TABLE 5:** Comparison based on consolidation‐to‐tumor ratio in patients receiving lobectomy for primary pulmonary adenocarcinoma with GGO component

	CTR < 62% (*N* = 258)	CTR ≧ 62% (*N* = 115)	*p*‐value
Age (years, mean ± SD)	59.73 ± 9.78	63.57 ± 9.26	0.0004
Gender (%)			0.289
Male	95 (36.8)	49 (42.6)	
Female	163 (63.2)	66 (57.4)	
Smoking status (%)	46 (17.8)	22 (19.1)	0.764
Family history (%)	36 (14.0)	7 (6.1)	0.028
Tumor size (cm, mean ± SD)			
GGO part	1.91 ± 0.90	2.49 ± 0.86	<0.0001
Solid part	0.56 ± 0.60	1.90 ± 0.66	<0.0001
Harvested lymph node (mean ± SD)			
Stations	4.4 ± 1.6	4.4 ± 1.4	0.871
Number	14.5 ± 7.8	15.4 ± 8.9	0.296
Pathological subtype (%)			<0.0001
Lepidic‐predominant	130 (50.4)	26 (22.6)	
Acinar‐predominant	100 (38.8)	71 (61.7)	
Papillary‐predominant	20 (7.8)	12 (10.4)	
Micropapillary‐predominant	4 (1.6)	4 (3.5)	
Solid‐predominant	4 (1.6)	2 (1.7)	
Pathological T stage (%)			<0.0001
Tis	24 (9.3)	0	
T1mi	38 (14.7)	1 (0.9%)	
T1a	26 (10.1)	3 (2.6)	
T1b	52 (20.2)	9 (7.8)	
T1c	24 (9.3)	15 (13.0)	
T2a	91 (35.3)	78 (67.8)	
T2b	2 (0.8)	8 (7.0)	
T3	1 (0.4)	1 (0.9)	
Pathological N stage (%)			0.001
N0	253 (98.1)	103 (89.6)	
N1	4 (1.5)	6 (5.2)	
N2	1 (0.4)	6 (5.2)	

Abbreviations: CTR, consolidation‐to‐tumor ratio; GGO, ground‐glass opacity; PET/CT, positron emission tomography/computed tomography.

## DISCUSSION

Lymphatic metastasis of lung adenocarcinoma is associated with poor prognosis and management for patients with lung cancer.^2^ According to the NCCN guidelines, PET/CT is recommended for the preoperative detection of regional lymphatic metastases or distant metastases in primary lung cancer.^2^ SUVmax of the lymph nodes exceeding 2.5 on PET/CT usually implies higher possibility of lymph node metastasis.^5^ However, many studies have pointed out that lung adenocarcinoma with a GGO component is less invasive with a relatively lower probability of lymphatic metastasis.^10^, ^11^, ^13^ Thus, whether PET/CT scan is still necessary for staging in these patients with GGO pulmonary nodules remains uncertain.

In this study, a total of 217 patients underwent lobectomy, and had a preoperative PET/CT scan. A total of 19 were found clinically to have lymph node metastasis, and only three out of them had pathologically proven lymph node metastasis, indicating a positive‐predictive value of 15.8%. On the other hand, among the 11 patients with lymph nodes metastasis, only three were clinically considered lymph node positive, indicating a sensitivity of 27.3%, lower than the report by Cerfolio et al.^5^ The sensitivity in detection of lymph node metastasis in lung cancer with GGO component was unsatisfactory, seeing that occult lymph node metastasis might be missed in preoperative setting. Conversely, at a cutoff value of 62%, CTR had 72.7% sensitivity for detection of lymph node metastasis.

Several reports have indicated CTR as a valuable prognostic factor. For example, Moon et al. reported solid predominant tumor as a significant factor for tumor recurrence in patients undergoing sublobar resection.^14^ In our study, all patients analyzed received lobectomy for a primary lung cancer, and all patients with lymph node metastasis had CTR > 60%. This further confirms the prognostic value of CTR. Some studies have suggested that a solid component indicates invasive adenocarcinoma;^9^, ^15^ in our study, eight of 11 patients with lymph node metastasis had an acinar component of tumor growth. Based on ROC curve and sensitivity tests, we derived a cutoff value of 62% for CTR. With this cutoff value, we determined that CTR was not inferior to main tumor SUVmax in terms of prediction of pathological nodal metastasis.

We adopted this cutoff value to classify all primary pulmonary adenocarcinoma with a GGO component receiving lobectomy into two groups. We found that patients with a CTR < 62% were younger and more had a positive family history of lung cancer. This phenomenon could be explained with increased early detection of lung cancer secondary to popularity of lung cancer screening. The stations and number of harvested lymph nodes were comparable. The group of patients with a CTR ≧62% had larger GGOs and solid part, and a significantly higher ratio of predominant papillary, micropapillary subtype, and more advanced T stage. On the other hand, the group of patients with a CTR <62% were found to have a less invasive histological component. This result was consistent with the study by Seidelman et al. which demonstrated that as the GGO component transforms into solid over time, it becomes more aggressive histologically.^9^

Multiple logistic regression analysis in our study revealed that younger patient age (<60 years) and main tumor SUVmax value were both significantly associated with lymph node metastasis, while the size of the tumor size was not. Koike et al. have also reported age <67 years as a risk factor for lymph node metastasis, but some other studies showed negative or insignificant results. ^8^, ^10^, ^16^ These confusing results may be as a result of a relatively conservative treatment policy for senile lung cancer patients, and therefore, any finding thus obtained should be interpreted carefully.

Our study found main tumor SUVmax > 2.5 as a risk factor for lymph node metastasis, but its predictive power was far less than previously reported,^14^ possibly because GGO demonstrated a relatively lower SUVmax value than solid nodules on PET/CT, thus influencing clinical judgment.

All the patients with pathological lymph node metastasis had a CTR ≧ 50%. Matsuguma et al. indicated that primary pulmonary adenocarcinoma with a GGO proportion greater than 50% is a favorable predictor of lymph node metastasis.^10^ Although in our study, there were still papillary‐predominant or micropapillary‐predominant adenocarcinoma in the CTR < 62% group, the results of negative lymph node metastasis was consistent with the study by Matsuguma et al.^10^

There are some limitations in this study. First, this was a retrospective study, in which some bias might exist during collection of clinical information. Second, the case number was relatively small and preoperative PET/CT examination was not available for every patient enrolled. Third, pure solid nodules were excluded from the study. Therefore, we were unable to investigate the prevalence of lymph node metastasis in the lung cancer patients with pure solid features.

In conclusion, in lung cancer patients with a GGO component, CTR was not inferior to main tumor SUVmax for the prediction of lymph node metastasis in a preoperative setting. PET/CT might not be necessary preoperatively in selected patients at low risk of incidence of lymph node metastasis. On the contrary, for lung cancer with a high CTR, a routine, invasive mediastinal staging should be performed. In the same way, major resection is mandatory to avoid locoregional recurrence. Further investigation is needed to elucidate the importance of CTR in patients with pulmonary GGO nodules.

## CONFLICT OF INTEREST

The authors have no conflicts of interest to declare, and there was no funding for this study.
